# Macro- and microscopic findings of ICG fluorescence in liver tumors

**DOI:** 10.1186/s12957-015-0615-5

**Published:** 2015-06-10

**Authors:** Shingo Shimada, Seiji Ohtsubo, Kazuhiro Ogasawara, Mitsuo Kusano

**Affiliations:** Department of Surgery, Japan Labor Health and Welfare Organization, Kushiro Rosai Hospital, 13-12, Nakazono-cho, Kushiro, Hokkaido 085-8533 Japan; Department of Oral and Maxillofacial Surgery, Japan Labor Health and Welfare Organization, Kushiro Rosai Hospital, 13-12, Nakazono-cho, Kushiro, Hokkaido 085-8533 Japan; Department of Surgery, Seiwa Memorial Hospital, 1-5-1-1, Kotoni, Nishi-ku, Sapporo, Hokkaido 063-0811 Japan

**Keywords:** Indocyanine green, Fluorescence imaging, Liver cancer, Paraffin-embedded tissue, Microscopic findings

## Abstract

**Background:**

Reports detailing microscopic observations of indocyanine green (ICG) fluorescence imaging (IFI) in hepatocellular carcinoma (HCC) and metastatic liver cancer are rare. We were able to perform macro- and microscopic IFI results in postoperative paraffin-embedded tissue samples and formalin-fixed specimens from liver tumors.

**Methods:**

Between April 2010 and March 2014, 19 patients with HCC or liver metastases of colorectal tumors underwent liver resection. ICG solution was injected into the peripheral vein from 14 to 2 days prior to operation. We observed liver tumor IFI during the laparotomy and IFI in resected liver sections using a photo dynamic emission (PDE) camera. The IFI of paraffin-embedded tissue samples was observed using a charge-coupled device (CCD) camera. Moreover, we microscopically performed tissue section IFI using a fluorescence microscope with an *ICG*-*B*-*NQF*.

**Results:**

We performed that IFI characteristics depended on tumor type macroscopically and microscopically. In normal liver tissue, fluorescence consistent with *the bile canaliculus* was observed. HCC had heterogeneous IFI, forming a total or partial tumor and rim pattern. In metastatic carcinoma, we performed that non-tumor cells in the marginal region showed fluorescence and tumor cells in the central region did not fluoresce.

**Conclusions:**

We confirmed that the variations of ICG fluorescence imaging patterns reflect different tumor characteristics in not only macroscopic imaging as previous reports but also microscopic imaging. Moreover, the ICG fluorescence method is useful for postoperative pathological detection of microscopic lesions in histopathological specimens. ICG fluorescence in paraffin-embedded tissue samples and formalin-fixed specimens is preserved in the long term.

## Background

Indocyanine green (ICG) combines with serum proteins in vivo and produces a fluorescence signal [1]. ICG fluorescence imaging (IFI) is increasingly being used in fluorescence-guided surgery, i.e., in breast [2], gastric [3, 4], and esophageal cancer [5] surgeries and vascular surgery [6]. Applications of IFI, including tattooing of liver segments [7, 8] and providing biliary tract contrast [9], have been reported in the liver surgery field. Some institutions have recently reported IFI macroscopic features in hepatocellular carcinoma (HCC) and metastatic liver tumors [10–13]. Liver tumors can be observed using fluorescence imaging and generally have heterogeneous fluorescence in the central region of the HCC [10, 11]. In contrast, metastatic liver tumors have a corona-like fluorescence pattern in the marginal region of the tumor [10, 12, 13]. Generally, IFI can be used for intraoperative tumor detection. However, there are few reports focused on postoperative IFI samples. Particularly, reports detailing microscopic IFI observations in HCC and metastatic liver tumors are rare.

In this study, we performed macro- and microscopic IFI characteristics in liver tumors using postoperative paraffin-embedded tissue and formalin-fixed specimens.

## Methods

Between April 2010 and March 2014, 19 patients with liver tumors underwent liver resection at the Japan Labor Health and Welfare Organization, Kushiro Rosai Hospital, Department of Surgery in Kushiro, Japan. This study was approved by the Ethics Committee of the Kushiro Rosai Hospital and performed according to the Helsinki Declaration guidelines. The clinicopathological characteristics and surgical data of the patients are shown in Table 1.

**Table 1:** Clinicopathological characteristics and macroscopic observations of IFI

Case	Age	Sex	Diagnosis	Location	Size (cm)	Number	ICGR15 (%)	AFP	PIVKA-II	CEA	CA19-9	Operation	Pathology and differentiation	vp	vv	im	Non-cancerous liver	IFI of surface	IFI of section
1	66	F	HCC	S6	3 × 3	1	11.0	2.2	16	NA	NA	Subsegmentectomy	Hepatocellular carcinoma—well	0	0	0	LC	Total tumor	Total, heterogeneously
2	79	F	HCC	S8	2 × 2	1	18.9	18.7	18	NA	NA	Subsegmentectomy	Hepatocellular carcinoma—well	0	0	0	LF	Total tumor	Total, heterogeneously
3	71	M	HCC	S6/7	8 × 8	2	4.4	10.4	NA	3.7	37.3	Posterior segmentectomy	Hepatocellular carcinoma—mod	0	0	0	NL	Total tumor	Total, heterogeneously
4	49	M	HCC	S7	1.5 × 1.4	1	7.9	32.9	25	NA	NA	Subsegmentectomy	Hepatocellular carcinoma—mod	0	0	1	LC	Total tumor	Total, heterogeneously
5	54	M	HCC	S7/8	3 × 3	1	14.7	90.8	95	4.2	0.2	Partial resection	Hepatocellular carcinoma—mod	0	0	0	LC	Total tumor	Total, heterogeneously
6	75	M	HCC	S8	2 × 2	1	17.3	1951.2	19	NA	NA	Partial resection	Hepatocellular carcinoma—mod	0	0	0	LF	Total tumor	Total, heterogeneously
7	80	F	HCC	S4	4.5 × 4.5	1	18.3	NA	6087	NA	NA	Partial resection	Hepatocellular carcinoma—por	1	1	1	CH	Total tumor	Partial, heterogeneously
8	78	M	HCC	S2/3	11 × 8	1	37.3	56934	363000	1.9	7.1	Lateral segmentectomy	Hepatocellular carcinoma—por	1	0	1	LF	Total tumor	Partial, heterogeneously
9	65	M	HCC	S7	1.5 × 1.5	1	11.7	2.7	42	4.5	NA	Partial resection	Hepatocellular carcinoma—por	0	0	0	LF	Total tumor	Rim
10	74	M	HCC	S5	6 × 6	1	8.2	6.4	83	2.1	3.9	Subsegmentectomy	Hepatocellular carcinoma—por	0	0	1	LC	Total tumor	Partial, heterogeneously
11	61	M	HCC	S8	1.4 × 1.4	1	9.9	45.5	23	NA	NA	Subsegmentectomy	Hepatocellular carcinoma—por	0	0	1	LC	Total tumor	Partial, heterogeneously
12	32	F	HCC	S5/6/7/8	15 × 12	1	7.0	1006.6	108	2.7	541.4	Right lobectomy	Hepatocellular carcinoma—unknown	0	0	0	NL	Total tumor	Rim
13	66	M	Metastasis of CRC	S6	2.5 × 2.5	1	16.1	NA	NA	2.7	6.3	Subsegmentectomy	Adenocarcinoma—mod	NA	NA	NA	NL	Total tumor	Rim
14	70	M	Metastasis of CRC	S6	2.5 × 2.5	1	11.5	NA	NA	3.1	2.2	Partial resection	Adenocarcinoma—mod	NA	NA	NA	NL	Rim	Rim
15	72	M	Metastasis of CRC	S5/8	4 × 4	1	9.7	4.1	17	6.6	8.9	Partial resection	Adenocarcinoma—mod	NA	NA	NA	NL	Rim	Rim
16	58	M	Metastasis of CRC	S6	0.8 × 0.8	1	16.0	NA	NA	2.4	44	Posterior segmentectomy	Adenocarcinoma—mod	NA	NA	NA	NL	Total tumor	Rim
17	45	M	Metastasis of CRC	S3	2.3 × 1.6	1	16.3	NA	NA	1.5	7	Lateral segmentectomy	Adenocarcinoma—mod	NA	NA	NA	NL	Total tumor	Rim
18	74	M	Metastasis of CRC	S6	1.5 × 1.5	1	5.0	3.9	NA	2.6	2.2	Partial resection	Adenocarcinoma—mod	NA	NA	NA	NL	Rim	Rim
19	66	M	Metastasis of CRC	S3, S4, S8	S3 4.8 × 4.5, S4 4.6 × 4.5, S8 1.7 × 1.5	3	19.1	NA	NA	7	886.6	Left lobectomy, partial resection	Adenocarcinoma—muc	NA	NA	NA	NL	Total tumor	Rim

ICG solution was injected into the peripheral vein from 14 to 2 days prior to the operation.

To evaluate the liver tissue, we also performed intraoperative ICG injection in several cases.

The ICG injection dose was 0.5 mg/kg. We observed the liver tumor IFI during the laparotomy and IFI in resected liver sections using a photo dynamic emission (PDE) camera (Hamamatsu Photonics, Hamamatsu). The IFI of paraffin-embedded tissue was obtained using a charge-coupled device (CCD) camera with a light-emitting diode at a 760-nm wavelength as the light emitter and a cut filter to filter light at wavelengths below 820 nm as the detector. Moreover, we microscopically observed tissue section IFI using a fluorescence microscope with an *ICG*-*B*-*NQF* (OPTO-LINE, Tokyo).

## Results

### Clinicopathological characteristics

A total of 19 patients were observed in this study. Of these patients, 12 patients had hepatocellular carcinoma, while seven patients had liver metastases of colorectal cancer (CRC). In HCC patients, the numbers of males and females were eight and four, respectively. Two cases were well differentiated, four cases had moderate differentiation and five cases had poor differentiation. One case had unknown differentiation. Four cases had partial resections performed, five cases had liver subsegmentectomies performed, two cases had liver segmentectomies performed, and one case had a hepatic lobectomy performed. Two cases had normal livers (NL), one case had chronic hepatitis (CH), four cases had liver fibrosis (LF), and five cases had liver cirrhosis (LC). In case No. 10, we were able to detect and resect by using IFI the main tumor and also small metastases, which were not identified with the preoperative imaging. In metastatic liver tumor cases, all patients were male and had partial resections performed. All cases had no cirrhosis or fibrosis (Table 1).

### Intraoperative observation

**Fig. 1:**
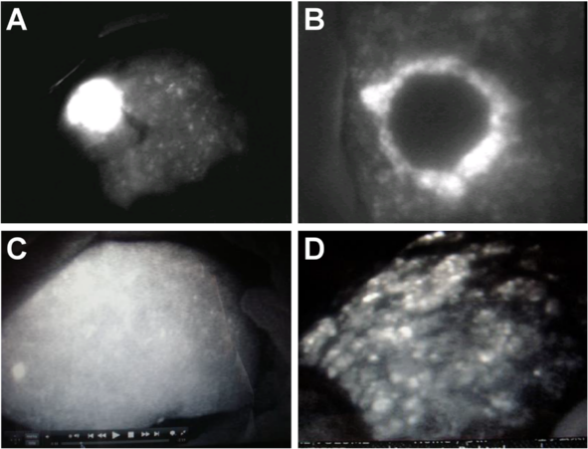
Intraoperative ICG fluorescence imaging (IFI). **a** Total tumor type (case 6). **b** Rim type (case 14). **c** Normal livers (non-cancerous part) had uniform fluorescence after intraoperative ICG injection (case 3). **d** Cirrhotic livers (non-cancerous part) had heterogeneous fluorescence, and the regenerated nodule was strongly fluorescent (case 5)

We were able to detect the targeted tumor by using IFI in all cases (Table 1). In the majority of cases, we were able to observe the whole tumor fluorescence (Fig. 1a), except in three cases with metastatic tumors derived from CRC that had rim fluorescence (Fig. 1b). Normal livers (non-cancerous sections) had uniform fluorescence after intraoperative ICG injection (Fig. 1c) while cirrhotic livers (non-cancerous sections) had heterogeneous fluorescence where the regenerated nodule strongly fluoresced (Fig. 1d). After ICG injection, we observed immediate emission and early washout in the normal liver. Conversely, we observed late emission and late washout in the cirrhotic liver as an aggravation of cirrhosis.

### Postoperative observation of liver sections

**Fig. 2:**
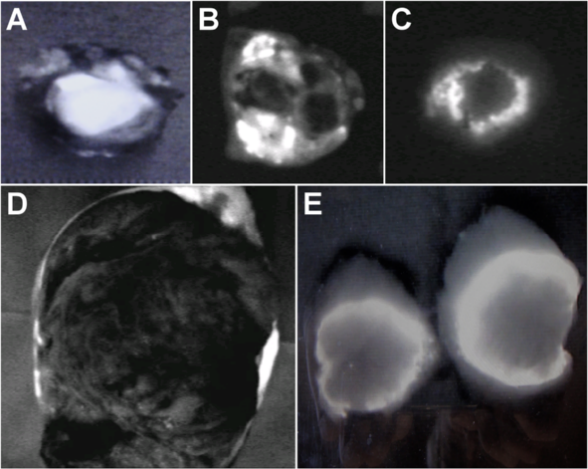
Postoperative observation of liver specimen sections. **a** Total tumor type (case 5). **b** Partial tumor type (case 10). **c** Rim type (case 9). **d** Rim type (case 12). **e** Rim type (case 14)

Six HCC cases had total tumor fluorescence emission (total tumor type), four cases had partial tumor fluorescence emission (partial tumor type), and two cases had rim-like fluorescence (rim type) in the liver sections (Table 1). All cases of well- or moderately differentiated tumors had total tumor type fluorescence (Fig. 2a). In five cases with poorly differentiated HCC, there were no tumors with total tumor type fluorescence, four cases with partial tumor type fluorescence (Fig. 2b), and one case (case 9) with rim type fluorescence (Fig. 2c). Case 12, which had an unknown HCC differentiation classification, had rim type fluorescence (Fig. 2d).

In cases of metastatic liver tumors derived from CRC, all cases had rim-like fluorescence at the marginal region of the tumor in the liver sections (Table 1) (Fig. 2e).

### Observations in paraffin-embedded tissue

**Fig. 3:**
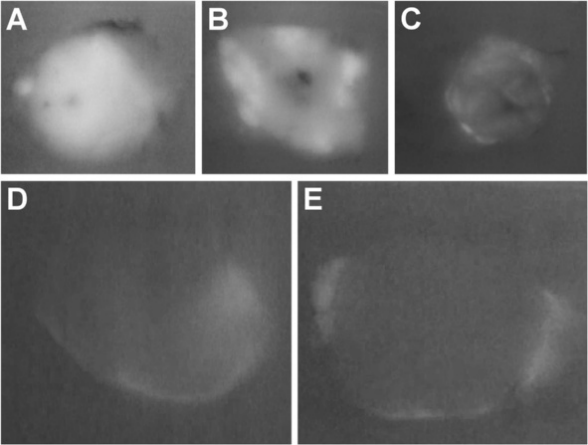
ICG fluorescence imaging (IFI) of paraffin-embedded tissue samples. ICG fluorescence was present in paraffin-embedded tissue samples. **a** Total tumor type (case 5). **b** Partial tumor type (case 10). **c** Rim type (case 9). **d** Rim type (case 12). **e** Rim type (case 14)

ICG fluorescence was not limited to fresh tissues but was also observable in paraffin sections (Fig. 3a–e). In the paraffin-embedded tissue samples, almost all cases had the same results as the sections; however, cases 7, 8, and 11 had different results compared with the liver sections. These samples had the rim type of IFI (Table 2).

**Table 2:** Paraffin block and microscopic observations of IFI

Case	Age	Sex	Diagnosis	Location	Size (cm)	Number	Non-cancerous live	IRI of paraffin block	IRI of microscopy	Time after operation
1	66	F	HCC	S6	3 × 3	1	LC	Total, heterogeneously	Cytosol of cancer cell	3 years, 8 months
2	79	F	HCC	S8	2 × 2	1	LF	Total, heterogeneously	Cytosol of cancer cell	2 years, 11 months
3	71	M	HCC	S6/7	8 × 8	2	NL	Total, heterogeneously	Cytosol of cancer cell	3 years, 4 months
4	49	M	HCC	S7	1.5 × 1.4	1	LC	Total, heterogeneously	Cytosol of cancer cell	3 years, 2 months
5	54	M	HCC	S7/8	3 × 3	1	LC	Total, heterogeneously	Cytosol of cancer cell	2 years, 9 months
6	75	M	HCC	S8	2 × 2	1	LF	Total, heterogeneously	Cytosol of cancer cell	1 year, 10 months
7	80	F	HCC	S4	4.5 × 4.5	1	CH	Rim	Nuclei of cancer cell and cytosol of non-cancer cell	1 year, 2 months
8	78	M	HCC	S2/3	11 × 8	1	LF	Rim	Nuclei of cancer cell and cytosol of non-cancer cell	3 years, 4 months
9	65	M	HCC	S7	1.5 × 1.5	1	LF	Rim	Nuclei of cancer cell and cytosol of non-cancer cell	3 months
10	74	M	HCC	S5	6 × 6	1	LC	Partial, heterogeneously	Cytosol of cancer cell	2 years, 4 months
11	61	M	HCC	S8	1.4 × 1.4	1	LC	Rim	Nuclei of cancer cell and cytosol of non-cancer cell	3 years, 7 months
12	32	F	HCC	S5/6/7/8	15 × 12	1	NL	Rim	Nuclei of cancer cell and cytosol of non-cancer cell	9 months
13	66	M	Metastasis of CRC	S6	2.5 × 2.5	1	NL	Rim	Cytosol of cancer cell	3 years, 2 months
14	70	M	Metastasis of CRC	S6	2.5 × 2.5	1	NL	Rim	Cytosol of cancer cell	2 years, 4 months
15	72	M	Metastasis of CRC	S5/8	4 × 4	1	NL	Rim	Cytosol of cancer cell	1 year, 8 months
16	58	M	Metastasis of CRC	S6	0.8 × 0.8	1	NL	Rim	Cytosol of cancer cell	2 years, 2 months
17	45	M	Metastasis of CRC	S3	2.3 × 1.6	1	NL	Rim	Cytosol of cancer cell	1 year, 4 months
18	74	M	Metastasis of CRC	S6	1.5 × 1.5	1	NL	Rim	Cytosol of cancer cell	1 year, 3 months
19	66	M	Metastasis of CRC	S3, S4, S8	S3 4.8 × 4.5, S4 4.6 × 4.5, S8 1.7 × 1.5	3	NL	Rim	Cytosol of cancer cell	4 months

We were able to observe tumor fluorescence in samples that were obtained 3 years and 8 months earlier in the paraffin-embedded tissue samples.

### Microscopic findings

**Fig. 4:**
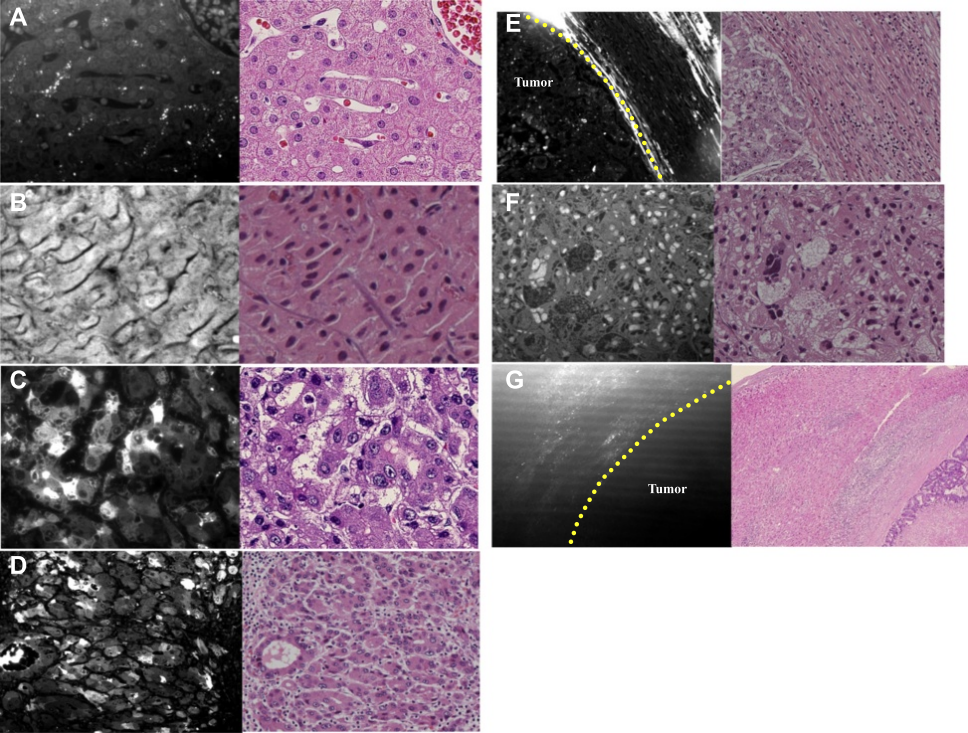
Microscopic findings from ICG fluorescence imaging (IFI) in tissue sections by using a fluorescence microscope with an *ICG*-*B*-*NQF* and HE staining of the adjacent section. **a** Normal liver; hepatocyte IFI was not strong; IFI in the bile canaliculus exhibited increased strength (case 3). **b** Cirrhotic livers; hepatocytes had stronger IFI than normal livers (case 5). **c** IFI was observed in the cytoplasm of cancer cells (case 9). **d** IFI was observed in the cytoplasm of cancer cells (case 4). **e** Tumor cells had heterogeneous fluorescence. We observed the IFI signal of cancer cells and of non-cancer cells surrounding cancer cells (case 12). **f** Nuclear IFI signal in cancer cells was observed (case 12). **g** IFI existed in the cytoplasm of non-cancer cells surrounding cancer cells (case 14)

Hepatocytes from cirrhotic livers had stronger IFI values than those from non-cirrhotic livers. While hepatocyte IFI values in non-cirrhotic livers were weaker than cirrhotic livers, the IFI values in the *bile canaliculus* in non-cirrhotic livers were higher than those in cirrhotic livers (Fig. 4a, b).

In hepatocellular carcinoma cases, tumor cells had heterogeneous fluorescence. Total or partial HCC tumor type paraffin-embedded tissue samples had cytoplasmic fluorescence as observed by ICG fluorescence microscopy (Fig. 4c, d). Furthermore, some HCC cases that had rim IFI had nuclear IFI in cancer cells and cytoplasmic IFI in the surrounding non-cancer cells (Fig. 4e, f). Metastatic cancers that had the rim type had cytoplasmic ICG fluorescence in non-cancer cells surrounding cancer cells (Table 2) (Fig. 4g).

## Discussion

In this study, we were able to perform the macroscopic and microscopic characteristics of different tumor types by using ICG fluorescence imaging (IFI). In normal hepatocytes, fluorescence consistent with the bile canaliculus was observed. In hepatocellular carcinoma, the IFI was heterogeneous and exhibited various patterns. In metastatic carcinoma, we observed that non-tumor cells in the marginal region showed fluorescence and tumor cells in the central region did not fluoresce.

ICG is generally used as a liver function assay [14]. Moreover, ICG, which is approved by the Food and Drug Administration, is safe and comparatively economical. ICG combines with serum proteins in vivo and fluoresces under excitation at 760–820 nm [1].

In animal experiments, ICG that was injected into the peripheral vein accumulated in the liver [15] demonstrating that ICG has a high affinity for the liver.

Within the liver surgery field, ICG fluorescence imaging applications have been reported for the detection of HCC or metastases derived from CRC [10, 11, 13] and pancreatic cancer [12], tattooing of the liver segment [7, 8], detection of biliary leakage [16, 17], and evaluation of hepatic congested areas [18] and vascular flow [19] after liver transplantation via contrast of the biliary tract. OATP1B3 and Na+ taurocholate cotransporting polypeptide (NTCP) have been demonstrated to transport ICG [20]. Eisai rats that lack functional MRP2 revealed that biliary ICG excretion is mediated by MRP2 [21].

Ishizawa et al. reported that well- or moderately differentiated HCC mostly displayed uniform or partial type fluorescence, poorly differentiated HCC mostly displayed rim type fluorescence, and metastatic tumors mostly displayed rim type fluorescence [10] in part because the degree of NTCP and OATP8 expression varied according to tumor differentiation [22]. Moreover, it was reported that there was a high false positive tumor detection rate in cirrhotic livers by using ICG fluorescence imaging [23]. In this study, these transporters were not evaluated, but the ICG fluorescence observations may be related to these transporters.

In this study, most tumors, particularly HCC tumors, appeared to be total tumor type from superficial observation. However, some cases had discrepancies between superficial observations and sectional observations.

All cases where the superficial observations were consistent with the sectional observations, except for the total tumor type, were cases where the tumor was exposed on the hepatic surface.

Although we observed that many IFI had total type fluorescence from the surface view, some tumor sections had partial or rim IFI. We believe that the IFI discrepancy between the surface and sections may be present in cases where the tumor is located far from the surface, resulting in unclear total IFI.

We observed consistent results between sections and paraffin-embedded tissues in most cases, including all metastatic tumor cases. However, the results were not consistent in three HCC cases. All these cases had poorly differentiated HCC. Poorly differentiated HCC cases had more variation in IFI results, i.e., the partial type or rim type, in comparison with well- and moderately differentiated HCC.

This result was consistent in sections and paraffin-embedded tissues. We believe that the cause of the discrepancy between sections and paraffin-embedded tissues is IFI tumor heterogeneity.

This would suggest that IFI tumor heterogeneity increases *as tumor differentiation fall*. We suggest that ICG is excreted more slowly in less differentiated cancer cells. ICG uptake and excretion differ depending on the cancer cells, and we are able to perform this “heterogeneity” using a fluorescence microscope.

Ishizawa et al. reported that HCC of the total or partial tumor type exhibited fluorescence within the cytoplasm and pseudoglands under ICG fluorescence imaging. Additionally, HCC or metastatic cancer with the rim type had ICG fluorescence in non-cancer cells surrounding cancer cells [22]. They suggested that the cause was the following: while ICG portal uptake ability was preserved, biliary excretion ability was reduced in differentiated HCC [22]. The biliary excretion ability of normal hepatocytes declined due to compression from the tumor in poorly differentiated HCC or metastatic cancer.

Consistent with these reports, in the present study, it was observed that the total or partial tumor type of HCC exhibited cytoplasmic fluorescence signal under ICG fluorescence microscopy, and metastatic cancers with the rim type exhibited ICG fluorescence in non-cancer cells surrounding the cancer cells. In some cases that exhibited rim IFI in poorly differentiated HCC or HCC of unknown differentiation status, there was nuclear IFI signal in cancer cells and cytoplasmic IFI signal in non-cancer cells surrounding the cancer cells.

Cancer cells may not fluoresce in metastatic liver cancer cases because cancer cells do not have the characteristics of hepatocytes. Macroscopic observations between metastatic liver cancer and HCC with the rim type were the same; however, microscopic observations between metastatic liver cancer and HCC with the rim type were somewhat disparate. While cancer cells did not exhibit IFI signal in metastatic liver cancers, the cancer cells exhibited IFI signal in different regions in poorly differentiated HCC or HCC of unknown differentiation status compared with other types. The exact cause of this phenomenon is elusive but may be related to the tumor characteristics derived from differentiation. Further investigation is necessary to clarify these observations.

In non-cancerous liver tissue, hepatocytes in cirrhotic livers had stronger IFI values than non-cirrhotic livers. However, there were stronger IFI values in *the bile canaliculus* in non-cirrhotic livers than cirrhotic livers. This result may be due to the early washout of ICG resulting from the preserved ICG-excretion ability of hepatocytes. After ICG injection, we observed immediate emission and early washout in the normal liver. Conversely, we observed late emission and late washout in cirrhotic livers as an aggravation of cirrhosis. Therefore, the regenerated nodule had an emission as strong as the tumor. In certain instances, there may not be a differentiation between the regenerated nodule and the tumor.

In this study, we successfully performed IFI at the cellular level using fluorescence microscopy with an *ICG*-*B*-*NQF*. This type of research is rare and is a useful method for progressing ICG fluorescence method research. However, the limitation of this study is the following. The period from surgery to observation was different about the paraffin blocks and microscopic sections because we retrospectively observed these samples. Then, we could not compare fluorescence intensity between three imaging techniques because the devices and condition between three imaging techniques were different.

In this study, we were able to observe fluorescence in a paraffin-embedded tumor tissue sample that was obtained more than 3 years earlier. Therefore, we can retrospectively evaluate tumor fluorescence. For example, we could retrospectively detect micro lesions in histopathological specimens using the ICG fluorescence method. It has been suggested that ICG, which is bound to intracellular proteins, is preserved in paraffin-embedded tissue. However, the precise mechanism of the ICG fluorescence retention is elusive. We did not investigate the optimal conditions for paraffin-embedded tissue to preserve fluorescence intensity. Further study is necessary to define the optimal conditions for preserving the fluorescence signal. However, we believe that discovering that ICG fluorescence in paraffin-embedded tissue samples can be retrospectively observed is very important.

## Conclusions

We confirmed that the variations of ICG fluorescence imaging patterns reflect different tumor characteristics in not only macroscopic imaging as previous reports [10, 22] but also microscopic imaging. Moreover, the ICG fluorescence method is also useful for postoperative pathological detection of micro lesions in histopathological specimens. ICG fluorescence in paraffin-embedded tissue samples and formalin-fixed specimens is preserved in the long term. However, further investigations are needed to clarify the reasons and optimal conditions for sample preservation.

## Abbreviations

CCD: charge-coupled device
CH: chronic hepatitis
CRC: colorectal cancer
HCC: hepatocellular carcinoma
ICG: indocyanine green
IFI: ICG fluorescence imaging
LC: liver cirrhosis
LF: liver fibrosis
MRP2: multidrug resistance associated protein 2
NL: normal liver
NTCP: Na+ taurocholate cotransporting polypeptide
OATP: organic anion transporting polypeptide
PDE: photo dynamic emission
